# Trends in the prevalence of self-reported diabetes in Brazilian capital cities and the Federal District, 2006–2014

**DOI:** 10.1186/s13098-016-0185-x

**Published:** 2016-10-13

**Authors:** Betine Pinto Moehlecke Iser, Álvaro Vigo, Bruce Bartholow Duncan, Maria Inês Schmidt

**Affiliations:** 1 Post Graduate Program in Epidemiology, School of Medicine, Universidade Federal do Rio Grande do Sul, Rua Ramiro Barcelos, 2600/414, Porto Alegre, RS 90035-003 Brazil; 2 Faculty of Medicine, Universidade do Sul de Santa Catarina, Tubarão, Brazil

**Keywords:** Linear models, Self-report, Diabetes trends, Behavioral Risk Factor Surveillance System, Health surveys

## Abstract

**Background:**

Diabetes is increasing globally, particularly in low and middle income countries, posing a great challenge to health systems. Brazil is currently ranked 4th in the world in terms of the absolute number of persons with diabetes. Our aim was to analyze the trend in self-reported diabetes prevalence between 2006 and 2014 in Brazilian adults.

**Methods:**

We used data from the national telephone survey—VIGITEL. Over 40,000 individuals from probabilistic sample of subjects ≥18 years old residing in 26 state capitals and the Federal District were interviewed per year in each location. Estimates were weighted to represent the surveyed population. We analyzed trends with a linear regression model. We adjusted prevalence with a probability predictive margins model, using as reference categories: men, 18–24 years, ≥12 years of schooling and lean/normal weight.

**Results:**

From 2006 to 2014, the overall prevalence increased from 5.5 to 8.0 %, a net rise of 0.26 %/year (P = 0.001). After adjustment for sex, age, schooling and BMI categories, the trend decreased only slightly to 0.25 %/year. Relatively greater adjusted increases were present in men (0.28 %/year), in those ≥65 years (0.52 %/year), with ≤8 years of schooling (0.33 %/year) and in those overweight (0.24 %/year). The most consistent upward trends were observed among men (coefficient of determination, R^2^ = 0.93), those with educational attainment of 0–8 years (R^2^ = 0.81), those > 65 years (R^2^ = 0.79) and those who were overweight (R^2^ = 0.75). There was no significant trend in diabetes prevalence for the obese. As expected, the prevalence of self-reported diabetes was always higher among those with greater age, less schooling, in women, and in those with obesity. Being obese was associated with having more than twice the prevalence of diabetes of those normal/underweight.

**Conclusions:**

Prevalence of self-reported diabetes in Brazilian adults has risen between 2006 and 2014, especially among those 65 years or older, even after taking into account the sociodemographic and nutritional changes during the period. Regardless of possible causes (higher incidence, increased diagnosis or decreased mortality), this increase in prevalence has enormous implications for the health system, representing >300,000 newly diagnosed cases of diabetes yearly requiring health care.

## Background

To halt the rise in the prevalence of diabetes is one of the most difficult non-communicable diseases (NCDs) voluntary goals stated by the nations and the World Health Organization [1–4]. The International Federation of Diabetes (IDF) has projected a 53 % rise in the prevalence of diabetes by 2035 from its 2014 estimate of 8.3 %, affecting mostly low and middle income countries [5].

The rise in diabetes prevalence, which initiated in the last century, has been accompanied by marked societal changes, notably aging of the population, urbanization and industrialization, as well as by changes in the ways that people eat and move, and by the marked global increases in overweight and obesity [1–3].

A recent systematic review involving over 4 million subjects aged 18 years or older and covering 146 countries and territories indicated that age-standardized diabetes prevalence increased from 4.3 to 9.0 % in adult men, and from 5.5 to 7.9 % in adult women, between 1980 and 2014, respectively. The rise is greater in low and middle income countries, both in terms of the number of individuals affected and the relative percent increase. Based on these findings, the authors estimated that, if post-2000 trends continue, the probability of achieving the global NCD target by 2025 is around 1 % [6].

Brazil, a large high-middle income country ranks among the top five with the largest number of individuals affected with diabetes [5, 6]. The purpose of this study is to analyze the recent trend (2006–2014) in the prevalence of self-reported diabetes in the 26 capital cities and the Federal District of Brazil, both before and after taking into account changes in population age structure and obesity indices which occurred during the covered period.

## Methods

We analyzed secondary data from Vigitel (Sistema de vigilância de fatores de risco e proteção para doenças crônicas por inquérito telefônico), the Brazilian behavior risk fator telephone survey. Since 2006, Vigitel furnishes yearly estimates of the prevalence of diabetes based on replies to the question “Have you been told by a physician that you have diabetes?” Vigitel conducts telephone interviews in probabilistic samples of the adult (aged 18 or over) residents of the 26 capital cities of Brazil and the Federal District possessing land line telephones. Annually, it performs 1500–2000 of these interviews in each of these capitals, reaching a total of more than 40,000 individuals each year.

Selection of participants was performed via two stage probabilistic sampling: (1) using lists of Brazil’s main land line telephone operators, a systematic sample of 5000 telephone numbers in each city was drawn, followed by an additional sampling of 25 repeat sub-samples of 200 numbers; (2) for each active residential number for which contact with a consenting adult resident was achieved, one resident aged 18 or over was randomly selected to participate in the interview.

Given the complex sampling scheme of Vigitel and the fact that, according to the 2010 Brazilian census, 61 % of residences in the capitals have a land line [7], the prevalences found were weighted so as to diminish differences between the covered population and that without land lines. These weighting factors are based on the design factor, determined as the inverse of the number of land lines and of adults in the residence and a post-stratification factor, which considers the sociodemographic distribution according to sex, age and educational attainment of the sample obtained and of the total population of the capital cities and Federal District, this latter obtained from the population census of 2010 and intercensus projections between 2000 and 2010 census. For estimates related to the overall sample considering all capitals, an additional factor is added—the ratio of the proportion of adults in a given capital to the total of adults in the whole sample [8].

For the analyses of temporal trends, the annual prevalences and their respective 95 % confidence intervals (95 % CI) were obtained for each strata of sex, age, educational attainment, and body mass index (BMI). BMI was calculated at weight/height^2^, being imputed in the case of missing values. Adjusted prevalences were then obtained using probability predictive margins with the Stata command Margins. Predicted values considered the mean of the distribution of each covariate in the population at the moment of each survey included in the trend analysis [9]. We used those having the lowest prevalences as the reference categories in these adjustments: male sex, age 18–24 years, educational attainment ≥12 years, and BMI <25 kg/m^2^ (underweight/eutrophic). All analyses were performed with Stata 12.0.

We then performed an additional trend analysis based on multiple linear regression using these adjusted prevalences and having calendar year as the independent variable. We investigated non-linear relationships by adding quadratic terms to the model. The model fit was assessed using residuals (residual × predicted plot, normality of residuals, and homogeneity of variances) and the coefficient of determination of the final models (R^2^ being ≥70 %). In the presence of heteroscedasticity, we used a robust estimator to calculate the standard error of the regression coefficients.

The variance inflation factor in the models used was never greater than 1.15, indicating the absence of relevant multi-collinearity. We adopted a significance level of 5 %.

## Ethics, consent and permissions

The Vigitel survey was approved by the Brazilian National Commission of Ethics in Research (CONEP), register number 13081/2008 as updated on June 26, 2013 (No. 355.590). These analyses were approved by the Ethics in Research Committee of the Hospital de Clinicas de Porto Alegre, on March 3, 2010 (No. 100056). Data, after removal of personal identifiers, are publically distributed and can be solicited at http://svs.aids.gov.br/bases_vigitel_viva/.

## Results

Table 1 shows the Vigitel’s degree of success of reaching selected landlines and interviewing participants, as well as sociodemographic and nutritional status characteristics of the sample obtained each year. The success in connecting with the selected landlines and conducting the interview ranged from 76.5 % (2009) to 64.8 % (2012). Refusal to respond once contact was made reduced considerably across the time span of the surveys (9.1 % in 2006 and 3.9 % in 2014). The yearly sample size in each city, which through 2011 was always greater than 54,000 adults interviewed, diminished starting in 2012 due to logistic difficulties, including the low quality of the telephone lists obtained. The yearly samples became older, gained educational attainment and showed greater BMI as the years progressed, reflecting changes occurring in the Brazilian population over this time period.

**Table 1:** Characteristics of the sample and measures of success in telephone sampling

	2006	2007	2008	2009	2010	2011	2012	2013	2014
	% or mean (±SD)								
Final sample (n)	54,369	54,251	54,353	54,367	54,339	54,144	45,448	52,929	40,853
Response rate (%)^a^	71.1	71.5	74.6	76.5	76.4	64.9	64.8	71.5	65.2
Refusals (%)	9.1	7.7	5.8	3.0	2.3	2.2	5.9	3.9	3.9
Women (%)	53.9	53.8	53.9	53.9	53.9	53.9	53.9	53.9	53.9
Age^b^	42.1 (16.5)	42.3 (16.2)	43.2 (16.5)	43.6 (16.5)	44.2 (16.7)	45.1 (16.9)	46.2 (17.2)	47.9 (17.6)	48.3 (17.6)
Age strata (%), years									
18–24	18.9	18.2	17.9	17.2	17.1	16.7	16.4	15.9	15.6
24–34	25.4	25.4	25.4	25.5	25.4	25.4	25.3	25.3	25.3
35–44	20.6	20.5	20.4	20.3	20.1	20.0	19.9	19.7	19.6
45–54	15.8	15.9	16.1	16.3	16.4	16.6	16.8	16.9	17.1
55–64	10.0	10.2	10.4	10.7	10.9	11.1	11.4	11.6	11.8
≥65	9.4	9.8	9.8	10.0	10.1	10.2	10.4	10.5	10.6
Education, years^b^	10.1 (4.5)	11.0 (5.2)	10.9 (4.9)	11.2 (5.0)	11.2 (5.0)	10.8 (4.9)	11.1 (5.0)	11.2 (5.0)	11.1 (5.1)
Educational attainment (%), years									
0–8	45.5	45.0	43.7	42.0	40.6	38.8	36.8	36.6	35.9
9–11	33.3	35.1	34.7	35.8	35.8	36.7	38.5	37.5	38.1
≥12	21.2	19.8	21.6	22.2	23.5	24.5	24.7	25.9	25.9
BMI (kg/m^2^)^b^	24.9 (4.5)	25.0 (4.6)	25.2 (4.6)	25.4 (4.7)	25.6 (4.8)	25.8 (4.9)	25.9 (4.9)	26.0 (4.9)	26.2 (4.9)
BMI categories (%)									
BMI < 25 kg/m^2^	57.3	56.7	55.1	54.0	51.8	50.9	49.0	49.2	47.5
25 kg/m^2^ ≤ BMI < 30 kg/m^2^	30.8	30.0	31.2	31.6	33.1	33.3	33.6	33.2	34.6
≥30 kg/m^2^	11.9	13.3	13.7	14.3	15.1	15.8	17.4	17.5	17.9

Vigitel 2006–2014

*BMI* body mass index

^a^Response rate: number of interviews done ÷ number of eligible phone lines selected (×100)

^b^Mean (standard deviation) without weighting

**Fig. 1:**
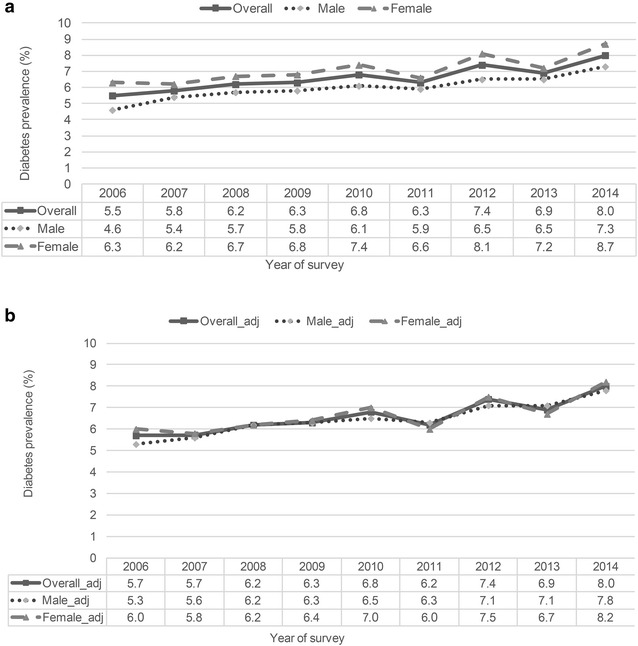
Prevalence of self-reported diabetes in adults of Brazilian capital cities and the Federal District, 2006–2014. **a** Crude. **b** Adjusted for age, educational achievement and BMI categories

Table 2 shows that the crude prevalence trended upward over the years studied, from 5.5 % in 2006 to 8.0 % in 2014, an increase of 0.26 % per year (P < 0.001). Increases (Fig. 1a) in this prevalence were seen for men (from 4.6 to 7.3 %) and for women (6.3–8.7 %). Table 2 also shows that, in absolute terms, the increase was greatest in older adults, notably from 35 to 44 years on, reaching a 0.57 %/year increase for those aged 65 or older. The absolute increase was greater in those with lesser schooling: from 2.8 to 5.1 % (0.25 %/year) for those with 9–11 years, and from 8.8 to 14.2 % (0.59 %/year) for those with less than 8 years. Among the BMI categories, the greatest absolute increase (0.22 %/year) was seen for those in the intermediate category between normalweight/underweight (BMI < 25 kg/m^2^) and obesity (BMI > 30 kg/m^2^), frequently referred to as overweight (25 kg/m^2^ ≤ BMI < 30 kg/m^2^).

**Table 2:** Crude prevalence of self-reported diabetes in adults (age 18 years and over) in the years 2006 and 2014, and annual increase over this period overall and in specific strata

Strata	2006	2014	Annual increase			
	% (95 % CI)	% (95 % CI)	%	(95 % CI)	P	R^2^
Total	5.5 (5.1–5.9)	8.0 (7.5–8.5)	0.26	0.16–037	0.001	0.83
Sex						
Men	4.6 (4.0–5.2)	7.3 (6.5–8.1)	0.26	0.18–0.34	<0.0001	0.89
Women	6.3 (5.7–6.8)	8.7 (8.0–9.4)	0.25	0.10–0.40	0.006	0.69
Age						
18–24	0.9 (0.5–1.3)	1.0 (0.4–1.6)	0.20	−0.06 to 0.09	0.57	0.05
25–34	1.1 (0.6–1.7)	1.6 (1.0–2.1)	0.01	−0.12 to 0.15	0.81	0.01
35–44	2.9 (2.3–3.6)	3.9 (3.0–4.9)	0.12	0.06–0.18	0.002	0.78
45–54	7.1 (6.0–8.2)	11.5 (9.9–13.0)	0.36	0.08–0.64	0.019	0.57
55–64	15.7 (13.6–17.8)	18.2 (16.2–20.1)	0.32	0.01–0.62	0.044	0.46
≥65	18.9 (17.0–20.8)	24.4 (22.4–26.5)	0.57	0.28–0.85	0.002	0.76
Educational attainment (years)						
≥12	2.8 (2.2–3.3)	3.7 (3.2–4.3)	0.12	0.00–0.23	0.051	0.44
9–11	2.8 (2.4–3.2)	5.1 (4.5–5.7)	0.25	0.11–0.39	0.005	0.70
0–8	8.8 (8.0–9.6)	14.2 (13.1–15.4)	0.59	0.40–0.79	<0.0001	0.88
BMI category						
<25 kg/m^2^	3.3 (3.0–3.7)	5.4 (4.8–6.0)	0.15	0.04–0.27	0.017	0.58
25 kg/m^2^ ≤ BMI < 30 kg/m^2^	6.9 (6.1–7.7)	8.6 (7.7–9.5)	0.22	0.10–0.34	0.003	0.73
≥30 kg/m^2^	12.8 (10.8–14.7)	14.0 (12.5–15.5)	0.14	0.17–0.46	0.32	0.14

Vigitel, Brazilian capital cities and the Federal District

Prevalences are weighted to reflect the sociodemographic distribution of the adult population of the sampled cities in each year of the survey

Significance level: 5 %

*BMI* body mass index

Table 3 shows prevalence trends overall and in sub-groups, now adjusting for changes over time in age, sex, educational attainment and BMI. Overall, the increase seen was similar, 0.25 %/year, from 5.7 % in 2006 to 8.0 % in 2014. Men again demonstrated a greater increase (from 5.3 to 7.8 %, 0.28 %/year) than women (from 6.0 to 8.2 %, or 0.23 %/year), although for both sexes the increase in prevalence was statistically significant (Fig. 1b). Estimates of change for those aged 18–34 years did not show a significant change, although the precision of estimates was lowest in this age range. From age 35 onward, the upward trend was statistically significant, being 0.11 %/year for those aged 35–44; 0.32 %/year for those aged 45–64; and 0.52 %/year for those 65 or older. The trend was statistically significant for all levels of educational attainment. For those with the least attainment, prevalences rose from 6.4 to 9.4 %, an increase of 0.33 %/year. The adjustment performed increased the rates seen in all three BMI categories, which now ranged from 0.16 %/year in the normalweight/underweight to 0.25 %/year in the obese, although the increase observed was only statistically significant for the overweight, in part due to the use of a robust variance estimator given the heteroscedasticity seen in the data among normalweight/underweight individuals. Analysis of residuals did not demonstrate aberrant points or lack of normality, and no collinearity was seen between variables. The most consistent upward trends were observed among men (coefficient of determination, R^2^ = 0.93), those with educational attainment of 0–8 years (R^2^ = 0.81), those >65 years (R^2^ = 0.79) and those who were overweight (R^2^ = 0.75).

**Table 3:** Adjusted prevalence of self-reported diabetes in 2006 and 2014 and trends over this time span overall and in specific strata

Strata	2006	2014	Annual increase			
	% (95 % CI)	% (95 % CI)	%	(95 % CI)	P	R^2^
Total	5.7 (5.3–6.0)	8.0 (7.6–8.5)	0.25	0.13–0.37	0.002	0.77
Sex						
Men	5.3 (4.6–5.8)	7.8 (7.0–8.6)	0.28	0.21–0.35	<0.001	0.93
Women	6.0 (5.5–6.5)	8.2 (7.6–8.8)	0.23	0.07–0.39	0.012	0.62
Age						
18–24	1.4 (0.7–2.0)	1.2 (0.5–2.0)	0.02	−0.09 to 0.14	0.708	0.02
25–34	1.4 (0.8–2.0)	1.8 (1.2–2.4)	0.02	−0.12 to 0.16	0.728	0.02
35–44	2.9 (2.3–3.4)	3.8 (2.8–4.7)	0.11	0.06–0.16	0.002	0.77
45–54	6.4 (5.4–7.3)	10.5 (9.1–11.9)	0.33	0.07–0.59	0.020	0.56
55–64	13.4 (11.6–15.2)	16.1 (14.3–17.9)	0.32	0.23–0.62	0.038	0.48
≥65	16.1 (14.3–17.8)	21.3 (19.3–23.3)	0.52	0.28–0.76	0.001	0.79
Educational attainment (years)						
≥12	4.1 (3.3–4.8)	5.4 (4.6–6.2)	0.16	0.02–0.31	0.033	0.50
9–11	4.6 (4.1–5.3)	7.1 (6.4–7.9)	0.26	0.08–0.43	0.010	0.63
0–8	6.4 (5.8–7.0)	9.4 (8.6–10.2)	0.33	0.19–0.47	0.001	0.81
BMI category						
<25 kg/m^2^	4.1 (3.7–4.5)	6.3 (5.6–6.9)	0.16	0.03–0.29	0.056	0.53
25 ≤ BMI < 30 kg/m^2^	6.0 (5.4–6.7)	7.9 (7.0–8.7)	0.24	0.12–0.37	0.003	0.75
≥30 kg/m^2^	9.6 (8.2–11.1)	11.8 (10.6–13.0)	0.25	0.02–0.53	0.063	0.41

Vigitel, Brazilian capital cities and the Federal District

Adjusted through multiple linear regression for changes over time in all other variables in the table

Prevalences are weighted to reflect the sociodemographic distribution of adult population of the sampled cities in each year of the survey

Level of significance: 5 %

*BMI* body mass index

## Discussion

The crude prevalence of self-reported diabetes in Brazilian capital cities increased from 5.5 % in 2006 to 8.0 % in 2014 (0.26 % per year; P < 0.001), both among men (4.6–7.3 %; 0.26/year) and women (6.3–8.7 %; 0.25/year). Taking into account changes over time during this period in diabetes risk factors, most notably obesity and aging, the upward trend in prevalence was altered only minimally, although the increase became more pronounced among men (0.28 %/year) than women (0.23 %/year). The increase was also more pronounced among the elderly (≥65 years), those with less education (≤8 years of schooling) and those overweight (≥25 kg/m^2^).

Such upward trends can, at least in part, result from wider access to diabetes diagnosis during this period. Advances in the care for diabetes have been made in the Brazilian national health system since the turn of the century, most notably the incorporation of diabetes care as a priority in primary care [10, 11], the increase in the number of specialty clinics, and the greater availability of services for the diagnosis and treatment of diabetes [12]. Recent surveys indicate that the percentage of adults ≥18 years referring never having done a glucose testing is small, between 11 and 24 % [13, 14]. Greater diagnosis as an explanation for at least part of the rise in self-reported diabetes in Brazil is supported by similar findings from the United States, in which nationally representative survey data from 1988 to 2012 document that a decrease in the proportion of unknown diabetes can explain part of the rise in diabetes prevalence in that country [15, 16]. Similar conclusions have been reached in Argentina [17].

Yet, other possibilities also need to be considered. First, it is possible the factors linked to an increased incidence of diabetes are at play, notably, aging of the population and the obesity epidemic. Although we adjusted for changes in age structure and obesity during the period investigated, our data do not permit investigation of the lag effects from similar changes over previous decades. Diabetes, after all, is a disease which develops slowly over decades, and Brazil has undergone rapid epidemiologic and nutrition transitions over the last several decades [18]. Nationally representative surveys from 1974–1975 to 2008–2009 show that the prevalence of obesity has been increasing steadily over this period, although the rate has slowed among women in the last two decades [19]. The Global Burden of Disease study has documented a similar rise in obesity globally, especially in more developed countries [4].

It is also possible that a decrease in mortality among those with diabetes—due to better treatment of people with the disease—contributes to the rise in prevalence, as this increase in life expectancy generates additional cases of diabetes. Cardiovascular mortality, the most important cause of death among those with diabetes, has decreased continuously over recent decades in Brazil [20, 21]. Analysis of mortality due to diabetes in Brazil show decreases, though small, over recent years, especially among women [22, 23].

The above-mentioned systematic review [6] showed increases in age-standardized diabetes prevalence in men and women similar to those we report, although they ascertained diabetes based on self-reported diabetes and fasting plasma glucose. The magnitude of the rise we observed is of great significance. Brazil, is a middle income country striving to control emergent and re-emergent infectious diseases while tackling the growing burden of non-communicable diseases. The increase in the prevalence of diabetes here reported represents a yearly increment of about 300,000 additional cases requiring diagnostic confirmation and continued care for diabetes over the years to come, which will undoubtedly result in a major increase in health care expenditures.

Limitations of our study merit mention. Our data are based on self-report, which misses a large fraction of the total cases of diabetes. Other than that, self-report of diabetes shows little disagreement with other sources of information [24–26]. Further, we are unable to classify these patients by their type of diabetes, although, previous analyses based on age at diagnosis suggest that the vast majority of the cases are of type 2 diabetes [13]. The use of just landline telephones to reach participants, and changing rates of response and refusal over time are additional limitations. However, Vigitel adjustments are performed to account for this potential bias and comparison of Vigitel data, once appropriately weighted to take into account these sampling issues, with those from household surveys do not show important differences in diabetes prevalence [27, 28]. Moreover, although we cannot generalize our findings to all Brazilian adults, a recent national survey [29] found a similar prevalence (6.5 vs. 6.9 % for Vigitel, in 2013) for the urban Brazilian population, which today represents over 85 % of the total population.

Strengths of our study include the size and breath of the Vigitel surveys, and their consistency in format over the 9 year period studied. The diagnostic criteria for diabetes have not changed over this period, other than those for gestational diabetes, which is not the subject at hand. Since 2006, Vigitel data have been collected by the same outsourced research survey firm, following specific protocols, with standard and frequent training of interviewers and quality control procedures such as recording of interviews for audit.

## Conclusion

We found a steady rise in the prevalence of self-reported diabetes over the period from 2006 to 2014, independent of changes in population age structure or obesity. This finding highlights how difficult it will be to halt the rise in diabetes prevalence and indicates that it is very unlikely that Brazil will achieve this voluntary NCD goal by 2025. Regardless of possible causes (higher incidence, increased diagnosis or decreased mortality), this increasing prevalence has enormous implications for the health system.

## Abbreviations

BMI: body mass index
CI: confidence interval
CONEP: National Commission of Ethics in Research
IDF: International Federation of Diabetes
NCDs: non-communicable diseases
WHO: World Health Organization
Vigitel: Vigilância de Fatores de Risco e Proteção para Doenças Crônicas por inquérito telefônico (Surveillance of risk and protective factors for Chronic Diseases by telephone survey)

## References

[1] Danaei G, Finucane MM, Lu Y, Singh GM, Cowan MJ, Paciorek CJ (2011). National, regional, and global trends in fasting plasma glucose and diabetes prevalence since 1980: systematic analysis of health examination surveys and epidemiological studies with 370 country-years and 2.7 million participants. Lancet.

[2] World Health Organization. Global status report on noncommunicable diseases 2014 (internet). Geneva: World Health Organization; 2014. p. 298. http://www.who.int/nmh/publications/ncd-status-report-2014/en/. Accessed 28 Jan 2015.

[3] International Diabetes Federation. IDF Diabetes Atlas (internet). 6th ed. Brussels: International Diabetes Federation; 2013. p. 160. https://www.idf.org/sites/default/files/EN_6E_Atlas_Full_0.pdf. Accessed 9 Oct 2015.

[4] Ng M, Fleming T, Robinson M, Thomson B, Graetz N, Margono C (2014). Global, regional, and national prevalence of overweight and obesity in children and adults during 1980–2013: a systematic analysis for the Global Burden of Disease Study 2013. Lancet.

[5] International Diabetes Federation. Diabetes atlas—IDF diabetes atlas update poster (internet). Brussels: International Diabetes Federation. 2014. https://www.idf.org/diabetesatlas. Accessed 9 Oct 2015.

[6] NCD Risk Factor Collaboration (NCD-RisC) (2016). Worldwide trends in diabetes since 1980: a pooled analysis of 751 population-based studies with 4.4 million participants. Lancet Lond Engl.

[7] Ministério do Planejamento Orçamento e Gestão, Brasil. Instituto Brasileiro de Geografia e Estatística (IBGE). Censo demográfico 2010—Características da população e dos domicílios. Resultados do universo (internet). Rio de Janeiro: IBGE; 2011. http://biblioteca.ibge.gov.br/visualizacao/periodicos/93/cd_2010_caracteristicas_populacao_domicilios.pdf. Accessed 11 Jun 2014.

[8] Ministério da Saúde. Secretaria de Vigilância em Saúde, Brasil. Departamento de Doenças e Agravos não transmissíveis e Promoção da Saúde. Vigilância de Fatores de Risco e Proteção para Doenças Crônicas por Inquérito Telefônico, Vigitel 2014. Brasília: Ministério da Saúde; 2015. p. 154. (Série G. Estatística e Informação em Saúde).

[9] Geiss LS, Wang J, Cheng YJ, Thompson TJ, Barker L, Li Y (2014). Prevalence and incidence trends for diagnosed diabetes among adults aged 20 to 79 years, United States, 1980–2012. JAMA.

[10] Viacava F (2010). Acesso e uso de serviços de saúde pelos brasileiros. RADIS.

[11] Ministério do Planejamento, Orçamento e Gestão, Brasil. Instituto Brasileiro de Geografia e Estatística (IBGE)., Brasil. Ministério da Saúde. Secretaria de Vigilância em Saúde. Pesquisa Nacional por amostra de domicílios (PNAD 2008). Um panorama da saúde no Brasil: acesso e utilização dos serviços, condições de saúde e fatores de risco e proteção à saúde: 2008. Rio de Janeiro: IBGE; 2010.

[12] Paim J, Travassos C, Almeida C, Bahia L, Macinko J (2011). The Brazilian health system: history, advances, and challenges. Lancet.

[13] Iser BPM, Malta DC, Duncan BB, de Moura L, Vigo A, Schmidt MI (2014). Prevalence, correlates, and description of self-reported diabetes in Brazilian Capitals—results from a telephone survey. PLoS One.

[14] Malta DC, Iser BPM, Chueiri PS, Stopa SR, Szwarcwald CL, Schmidt MI (2015). Cuidados em saúde entre portadores de diabetes mellitus autorreferido no Brasil, Pesquisa Nacional de Saúde, 2013. Rev Bras Epidemiol.

[15] Menke A, Rust KF, Fradkin J, Cheng YJ, Cowie CC (2014). Associations between trends in race/ethnicity, aging, and body mass index with diabetes prevalence in the United States: a series of cross-sectional studies. Ann Intern Med.

[16] Menke A, Casagrande S, Geiss L, Cowie CC (2015). Prevalence of and trends in diabetes among adults in the United States, 1988–2012. JAMA.

[17] Rubinstein A, Gutierrez L, Beratarrechea A, Irazola VE (2014). Increased prevalence of diabetes in Argentina is due to easier health care access rather than to an actual increase in prevalence. PLoS One.

[18] Instituto Brasileiro de Geografia e Estatísticas—IBGE. Indicadores sociodemográficos e de saúde no Brasil (internet). Rio de Janeiro: IBGE; 2009. p. 154. (Estudos e pesquisas. Informação demográfica e socioeconômica). http://www.ibge.gov.br/home/estatistica/populacao/indic_sociosaude/2009/. Accessed 16 Jun 2016.

[19] Conde WL, Monteiro CA (2014). Nutrition transition and double burden of undernutrition and excess of weight in Brazil. Am J Clin Nutr.

[20] Schmidt MI, Duncan BB, e Silva GA, Menezes AM, Monteiro CA, Barreto SM (2011). Chronic non-communicable diseases in Brazil: burden and current challenges. Lancet.

[21] Ribeiro ALP, Duncan BB, Brant LCC, Lotufo PA, Mill JG, Barreto SM (2016). Cardiovascular health in Brazil: trends and perspectives. Circulation.

[22] Malta DC, de Moura L, do Prado RR, Escalante JC, Schmidt MI, Duncan BB (2014). Mortalidade por doenças crônicas não transmissíveis no Brasil e suas regiões, 2000 a 2011. Epidemiol E Serviços Saúde.

[23] Schmidt MI, Duncan BB, Ishitani L, da Conceição Franco G, de Abreu DMX, Lana GC (2015). Trends in mortality due to diabetes in Brazil, 1996–2011. Diabetol Metab Syndr.

[24] Francisco PM, Barros MB, Segri NJ, Alves MC, Cesar CL, Malta DC (2011). Comparison of estimates for the self-reported chronic conditions among household survey and telephone survey-Campinas (SP), Brazil. Rev Bras Epidemiol.

[25] Huerta JM, Tormo MJ, Egea-Caparros JM, Ortola-Devesa JB, Navarro C (2009). Accuracy of self-reported diabetes, hypertension and hyperlipidemia in the adult Spanish population. DINO study findings. Rev Esp Cardiol.

[26] Jackson JM, DeFor TA, Crain AL, Kerby TJ, Strayer LS, Lewis CE (2014). Validity of diabetes self-reports in the women’s health initiative. Menopause.

[27] Bernal RT, Malta DC, de Araujo TS, Silva NN (2013). Inquérito por telefone: pesos de pós-estratificação para corrigir vícios de baixa cobertura em Rio Branco, AC. Rev Saúde Pública.

[28] Bernal RT, Malta DC, Morais Neto OL, Claro RM, Mendoça BC, Oliveira AC (2014). Vigitel—Aracaju, Sergipe, 2008: the effects of post-stratification adjustments in correcting biases due to the small amount of households with a landline telephone. Rev Bras Epidemiol Braz J Epidemiol.

[29] Iser BP, Stopa SR, Chueiri PS, Szwarcwald CL, Malta DC, Monteiro HO (2015). Prevalência de diabetes autorreferido no Brasil: resultados da Pesquisa Nacional de Saúde 2013. Epidemiol E Serviços Saúde.

